# Obesity-induced nucleosome release predicts poor cardio-metabolic health

**DOI:** 10.1186/s13148-019-0797-8

**Published:** 2019-12-31

**Authors:** Oriana Lo Re, Andrea Maugeri, Jana Hruskova, Juraj Jakubik, Jan Kucera, Julie Bienertova-Vasku, Jude A. Oben, Lukas Kubala, Adela Dvorakova, Milan Ciz, Manlio Vinciguerra

**Affiliations:** 1 International Clinical Research Center, St Anne’s University Hospital, Brno, Czech Republic; 2 Department of Biology, Faculty of Medicine, Masaryk University, Brno, Czech Republic; 3 Department of Medical and Surgical Sciences and Advanced Technologies “GF Ingrassia”, University of Catania, Catania, Italy; 4 Research Centre for Toxic Compounds in the Environment (RECETOX), Faculty of Science, Masaryk University, Brno, Czech Republic; 5 Department of Pathological Physiology, Faculty of Medicine, Masaryk University, Brno, Czech Republic; 6 Institute for Liver and Digestive Health, Division of Medicine, University College London (UCL), London, UK; 7 Institute of Biophysics, Academy of Sciences of the Czech Republic, 61265 Brno, Czech Republic; 8 Department of Animal Physiology and Immunology, Institute of Experimental Biology, Masaryk University, Brno, Czech Republic

**Keywords:** Liquid biopsy, Nucleosome, Epigenetics, Metabolic health, Cardiovascular disease

## Abstract

**Objective:**

While circulating nucleosome levels are high in obese mouse models, it is unknown where these nucleosomes originate from and whether they are a marker of cardio-metabolic health in humans. Here, we aimed to determine whether an association exists between circulating nucleosomes and the risk of developing obesity, metabolic syndrome (MetS) and/or a dysfunctional cardiovascular performance.

**Methods:**

We randomly selected 120 participants of the Kardiovize Brno 2030 study across three BMI strata: BMI 18–25, 25–30, and > 30. We assessed the association between circulating nucleosome levels and the risk of obesity, MetS, and poor cardiovascular health. We then cultured human neutrophils, adipocytes, and hepatoma cells to study nucleosome origins in a fat-rich environment.

**Results:**

Circulating nucleosome levels positively correlated with BMI (*R* = 0.602, *p* < 0.05), fatty liver index (*R* = 0.622, *p* < 0.05), left ventricular mass (*R* = 0.457, *p* < 0.05), and associated with MetS (*p* < 0.001) and poor cardiovascular health (*p* < 0.001). Incubating neutrophils with 1–10 μM free fatty acids triggered nucleosome production without concomitant cell death. Nucleosomes were not produced during pre-adipocyte differentiation or upon incubation of hepatic cells with palmitic acid.

**Conclusions:**

Neutrophils are a bona fide source of circulating nucleosomes in an obesogenic environment and in overweight/obese patients. High nucleosome levels are associated with MetS and cardiovascular performance, and might represent novel candidate biomarkers for cardio-metabolic health.

## Introduction

The nucleosome—the basic repeating unit of chromatin—was described > 40 years ago as DNA wrapped by an octameric core of histones [1]. Nucleosomes allow genome compaction and protection in the cell nuclei, and their composition and post-translational modifications regulate gene expression [2]. Cell-free DNA, histones, and nucleosomes are released into the blood stream upon cell death, both in health and disease. Interestingly, intact nucleosome levels in the circulation are elevated in several cancers and in acute conditions such as stroke, trauma, and sepsis [3, 4]. Consequently, the use of circulating free DNA and nucleosomes from human plasma as a standard noninvasive diagnostic tool is rapidly gain momentum [3, 4]. Indeed, chromosome abnormalities are routinely detected by assaying fetal DNA in the mother’s blood [5]. Furthermore, a strong diagnostic and prognostic performance for circulating nucleosomes has been reported for pancreatic [6], lung [7], colorectal [8], and breast cancers [9]. In terms of the diagnostic value, anti-nucleosome antibodies have been shown to be > 2-fold more sensitive compared to anti-DNA antibodies in the detection of autoimmune diseases, but less specific [10]. The potential of circulating nucleosomes to serve as biomarkers, or “liquid biopsies” is, therefore, a promising area of research for early cancer detection and monitoring treatment responses. From a mechanistic view point, recent approaches aiming at mapping nucleosome occupancy have helped to identify the tissue of origin of circulating free DNA in healthy individuals [11]; however, it remains a challenge to identify the tissue of origin(s) of circulating nucleosomes.

Metabolic syndrome (MetS) is a cluster of risk factors for cardiovascular disease and type 2 diabetes mellitus (T2DM). MetS risk factors include obesity (particularly central adiposity), hyperlipidemia, insulin resistance, and hypertension [12]. MetS doubles the risk of cardiovascular disease (CVD), more than triples the risk of cardiovascular mortality, and elevates T2DM risk by approximately five-fold. The association between MetS and CVD development is believed to be multi-factorial, where insulin resistance, oxidative stress, low-grade inflammation, and vascular maladaptation all make a major contribution [13, 14]. Furthermore, available evidence from epidemiologic, experimental, and clinical studies supports the emerging hypothesis that MetS might be an important etiologic factor for the development and progression of certain types of cancer and overall cancer mortality [15, 16]. Today, MetS constitutes a real pandemic, affecting one-third of adults in the USA [17, 18].

A study by Nishimoto et al. demonstrated that obesity increased cell-free DNA and nucleosome levels in the plasma of mice fed a high-fat diet [19]. Using the same obesogenic mouse model, Revelo et al. showed that circulating DNA-targeting pathways promoted inflammation and MetS; this finding also correlated with increased circulating levels of histone H3 [20]. In both humans and rats displaying acute pancreatitis, a life-threatening inflammatory condition, obesity or high body mass index (BMI) promotes the release of extracellular nucleosomes into the blood stream [21]. Despite these compelling data, it is unknown whether circulating nucleosomes might function as markers of cardio-metabolic health and MetS in humans in the absence of acute or severe illnesses, such as cancer.

To analyze the complex association of biological and behavioral risk factors with metabolic disturbances and CVD, we recently designed the Kardiovize Brno 2030 study, which recruited 2160 randomly selected residents from the urban population of Brno, Czech Republic [22, 23]. Previous results from this study suggested that altering meal frequency might be a potential preventive strategy against weight gain and CVD risk [24], that high adherence to a prudent dietary pattern (rich in cereals, fish, fruit, and vegetables) was associated with lower odds of abdominal obesity and MetS [18], and that consumption of antioxidants protect from subclinical atherosclerosis in a gender-specific manner [25]. In the current cross-sectional analysis of the Kardiovize Brno 2030 cohort, we aimed to determine whether an association exists between circulating nucleosomes and the risk of developing obesity, MetS, and/or a dysfunctional cardiovascular performance. Using primary and immortalized human cell cultures, we provide preliminary mechanistic evidence on the origin of circulating nucleosomes in the frame of an obesogenic-like environment.

## Materials and methods

### Study design

A baseline examination of the Cardiovision (Kardiovize) Brno 2030 study was completed in 2014, with planned prospective follow-up every 5 years until 2030 [22]. The study protocol was approved by the Ethics Committee of St Anne’s University Hospital, Brno, Czech Republic (reference 2 G/2012), with amendment: 2G/2012-AM (14.11.2018), in accordance with the Declaration of Helsinki, and all participants provided informed consent to participate in the study. All eligible participants completed a physical examination, with assessment of anthropometric, biochemical, and echocardiographic parameters. BMI (calculated as weight in kg divided by height in m^2^) was used to classify patients as normal weight (BMI 18–25), overweight (BMI 25–30), or obese (BMI > 30). Blood pressure was measured using a mercury sphygmomanometer (Baumanometer, W.A. Baum, Co., Inc., USA) and used to define hypertension (blood pressure ≥ 140/90 mmHg). The present study population consisted of 120 randomly selected subjects from the Kardiovize cohort, divided as follows: 46 participants with BMI 18–25 kg/m^2^ (normal weight), 34 participants with BMI 25–30 kg/m^2^ (overweight), and 40 participants with BMI ≥ 30 kg/m^2^ (obese).

### Physical examination and laboratory analyses

Physical examination was performed by trained professionals and in accordance with standardized and validated protocols [26]. In brief, height and weight were measured to the nearest 1 cm and 1 kg, respectively, using a medical digital scale with meter (SECA 799; SECA, GmbH and Co. KG, Hamburg, Germany). Waist circumference was measured to the nearest 1 cm by manual tape measurement, and central obesity was defined according to the World Health Organization criteria [27]. Biochemical analyses were performed on 12-h fasting blood samples using a Modular SWA P800 analyzer (Roche, Basel, Switzerland). Total cholesterol, triglycerides, and glucose levels were measured by the enzymatic colorimetric method (Roche Diagnostics GmbH, Germany); HDL-cholesterol was measured using the homogeneous method for direct measuring without precipitation (Sekisui Medical, Japan). For triglycerides < 4.5 mmol/l, the LDL-cholesterol level was calculated according to the Friedewald equation. For triglycerides > 4.5 mmol/l, the LDL-cholesterol level was calculated using the homogeneous method for direct measuring (Sekisui Medical, Japan). The Fatty Liver Index (FLI), an accurate predictor of hepatic steatosis in the general population, was calculated from serum triglycerides, BMI, waist circumference, and GGT, and categorized as previously described [28, 29].

### Definition of cardio-metabolic health

According to the International Diabetes Federation [12], we defined metabolic health if fewer than two of the following criteria were present: (i) systolic/diastolic blood pressure ≥ 130/85 mmHg or use of antihypertensive drug; (ii) triglycerides level ≥ 150 mg/dl; (iii) HDL-cholesterol level < 40 mg/dl in men or < 50 mg/dl in women or use of lipid-lowering drugs; and (iv) glucose level ≥ 100 mg/dl or use of antidiabetic drug. Based on this definition, participants were classified as metabolically healthy or metabolically unhealthy.

According to the American Heart Association (AHA) [30, 31], cardiovascular health (CVH) score was computed as the sum of seven metrics (i.e., BMI, healthy diet, physical activity level, smoking status, blood pressure, blood glucose, and total cholesterol). Each metric was scored from 0 to 2 (0 = poor, 1 = intermediate, and 2 = ideal) and thus the overall CVH score ranged from 0 to 14 [30, 31]. Poor CVH status was defined as having at least one of seven metrics at poor level [32].

### Echocardiography

Transthoracic echocardiography was performed with a GE-Vingmed Vivid E9 device (GE Vingmed Ultrasound AS, Horten, Norway) using a 1,5-4,6 MHz sector transducer. Images of the subcostal projections were captured during quiet breathing or at the end of expiration while the patient was in a left lateral decubitus or supine position. An electrocardiogram (ECG) was recorded and displayed simultaneously, and analysis was performed using EchoPAC PC software version 113. Chamber qualification and Doppler analyses were assessed according to the American Society of Echocardiography criteria [33–35].

### Assessment of circulating nucleosomes

Serum samples taken for nucleosome measurements were handled as previously described [22]. Circulating nucleosomes were assayed using commercially available ELISA kits (nucleosomes: Cell Death Detection ELISAPLUS, Roche, Mannheim, Germany). Global histone H3K4me3 levels were assessed by colorimetric assay using an EpiQuik™ Global Tri-Methyl Histone H3-K4 Quantification Kit (EpiGenTek, Lab Mark a.s., Prague, Czech Republic), according to manufacturer’s instructions.

### Cell cultures

HepG2 cell lines were purchased from the American Type Culture Collection (ATCC) and cultured in Dulbecco’s modified Eagle’s medium (1×) supplemented with 10% fetal bovine serum, with 1% penicillin/streptomycin [36].

Human multipotent adipose-derived stem (hMADS) cells were established and characterized as previously described [37]. hMADS cells were cultured according to a standard protocol [38]. The medium used for routine maintenance and the cultivation/differentiation protocol was similar to the hMADS cells.

Simpson-Golabi-Behmel syndrome (SGBS) preadipocyte cells (provided by Dr. Wabitsch) were established as previously described [39]. SGBS cell cultivation and differentiation was performed according to a standard protocol [40]. The cell culture supernatants were collected at the indicated times.

### Neutrophil isolation and assays

Whole blood samples were collected by venipuncture from healthy volunteers into tubes containing sodium citrate (100 μl of 3.8% citrate per 1 ml of blood). All patients provided informed consent and studies were conducted in accordance with the Helsinki Declaration and with approval of the Local Ethic Committee. For neutrophil isolation, the samples first underwent dextran (3%) sedimentation (blood/dextran ratio was 2/1) for 40 min at room temperature. Then, the leukocyte rich buffy coat was layered over Histopaque 1077 (Sigma-Aldrich, US) and centrifuged at 390×*g* for 30 min without brake. The remaining erythrocytes were removed by hypotonic hemolysis. The cell pellet was washed with PBS and the final suspension of neutrophils contained > 96% viable cells (CASY, Roche Diagnostics GmbH, Germany).

All neutrophils were used immediately after isolation: first, the neutrophils were incubated with palmitic acid (between 10 nM and 10 μM; Sigma-Aldrich, US) for 4 h. The chemiluminescence of the isolated neutrophils was then evaluated in a 96-well plate luminometer LM-01 T (Immunotech, Czech Republic) to determine reactive oxygen species production. Each reaction mixture consisted of 100,000 neutrophils per sample (treated with palmitic acid) diluted in HBSS and 1 μM luminal to a final volume of 250 μl. The samples were measured at 37 °C for 30 min. The data were acquired as peak values of a chemiluminescence signal.

Neutrophil supernatants were collected to determine lactate dehydrogenase (LDH) activity. An LDH release assay was performed using a Cytotoxicity Detection Kit^PLUS^ (LDH) (Roche Diagnostics GmbH, Germany), following the manufacturer’s protocol. CD11b marker expression was determined in neutrophil samples after 4 h incubation with palmitic acid by FACSVerse (BD Biosciences, US) using a CD11b antibody (Sony Biotechnology Inc., US).

### Statistical analyses

All statistical analyses were conducted using GraphPad Prism (version 6.0, GraphPad Software, USA) or SPSS Statistics software (version 22.0, IBM Corporation, USA). The Kolmogorov-Smirnov test was first used to test the normality of continuous variables before further analyses. A Spearman’s correlation analysis was performed to determine the correlation between continuous variables and nucleosomes levels. Continuous variables underlying a skewed distribution were compared using the Mann-Whitney *U* or Kruskal-Wallis tests. Continuous variables with normal distribution were compared using Student’s *t* test. The ability of serum circulating nucleosomes to discriminate patients with severe obesity, poor metabolic health, or CVH was assessed by receiver-operating characteristic (ROC) curve analysis. This analysis is a powerful tool in diagnostic testing, in which the test characteristics of sensitivity and specificity are relevant to discriminate diseased versus non-diseased conditions [41]. Accordingly, the area under the curve (AUC) and 95% confidence intervals (CIs) were calculated to assess the performance (sensitivity and specificity) of the test for each value of serum circulating nucleosomes. To determine the optimal threshold of serum circulating nucleosomes suitable to identify patients with poor metabolic health or CVH, the point on the ROC curve with the shortest distance value from the top left corner (point: 0,1) was calculated using the formula [(1 – sensitivity)^2^ + (1 – specificity)^2^]. All statistical tests were two-sided, and *p* values < 0.05 were considered statistically significant.

## Results

### Association of serum nucleosome levels with obesity and metabolic health

**Fig. 1:**
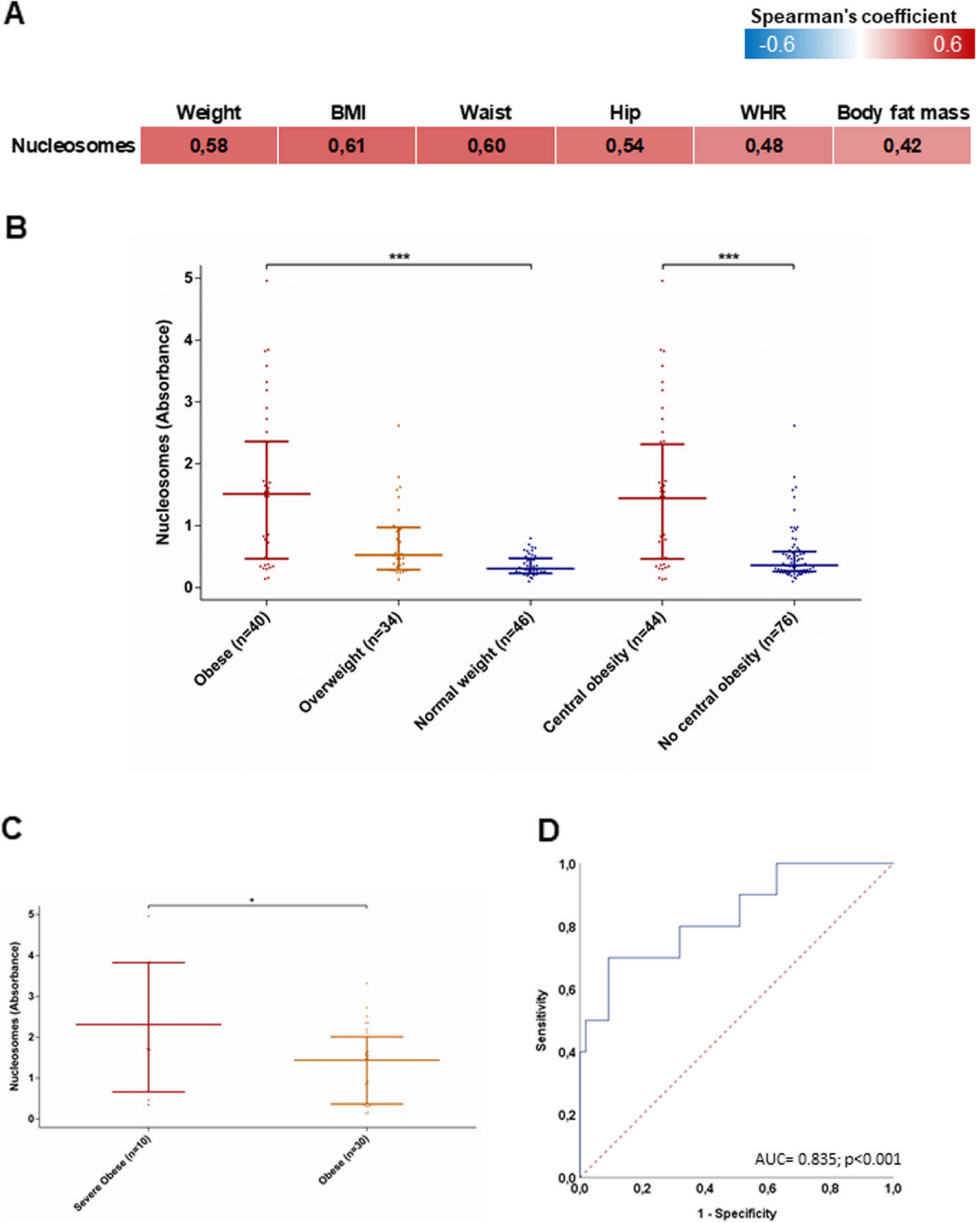
Association between circulating nucleosome levels, anthropometric measures and body mass index (BMI) categories. Circulating nucleosomes are assayed by ELISA in Kardiovize participants (*n* = 120) and expressed as absorbance. **a** Correlation matrix between circulating nucleosome levels and anthropometric measures. The data are reported as the Spearman’s correlation coefficient and those with a *p* value < 0.05 are indicated in bold. **b**, **c** Comparison of circulating nucleosome levels between BMI categories. The data represent the median and interquartile range and are compared using the Mann-Whitney *U* or Kruskal–Wallis tests; ***p < 0.001. (D) ROC curve showing the ability of serum circulating nucleosomes to discriminate patients with severe obesity. *BMI* body mass index, *WHR* waist to hip ratio

We randomly selected 120 participants of the Kardiovize cohort according to their BMI to assess serum nucleosome levels (Table 1 and Additional file 1: Table S1 show their cardio-metabolic and echocardiographic parameters according to BMI categories). We first demonstrated that serum nucleosome levels are positively correlated with anthropometric measures, including weight, BMI, waist and hip circumferences, WHR, and fat mass (*p* values < 0.001; Fig. 1a). Accordingly, serum nucleosome levels increased across BMI categories (*p* < 0.001), with higher levels in the presence of central obesity (*p* < 0.001; Fig. 1b). We also observed that participants with severe obesity (BMI ≥ 35 kg/m^2^) exhibited higher serum nucleosome levels than those with first class obesity (BMI 30–34.9 kg/m^2^) (*p* = 0.034; Fig. 1c). Notably, ROC curve analysis demonstrated that serum nucleosome levels could discriminate patients with severe obesity with an AUC of 0.835 (95% CI = 0.692–0.977; *p* < 0.001; Fig. 1d). According to the definition of the minimum distance on the ROC curve from the (0, 1) point, the cut-off value of 1.688 serum nucleosome levels gave optimal detection of subjects with super obesity (specificity = 90.9%; sensitivity = 70.0%).

**Table 1:** Characteristics of Kardiovize participants according to BMI categories

Characteristics	Normal weight (*n* = 40)	Overweight (*n* = 40)	Obese (*n* = 40)	*p* value
Age, years	44.0 (16.0)	48.0 (19.5)	49.0 (16.0)	0.393
Sex (% male)	54.3%	44.1%	50.0%	0.664
Waist circumference, cm	75.0 (14.0)	88.5 (11.0)	107.0 (15.0)	< 0.001
Hip circumference, cm	94.0 (5.0)	103.0 (5.0)	115.0 (12.0)	< 0.001
WHR	0.79 (0.13)	0.86 (0.12)	0.94 (0.10)	< 0.001
Body fat mass, %	19.0 (10.0)	25.9 (13.0)	38.1 (14.6)	< 0.001
Systolic blood pressure, mm Hg	112.5 (12.0)	119.8 (19.1)	125.0 (18.5)	< 0.001
Diastolic blood pressure, mm Hg	76.5 (11.5)	81.0 (12.3)	83.5 (10.0)	< 0.001
Total cholesterol, nmol/L	5.3 (1.7)	5.8 (1.3)	4.8 (1.2)	0.011
HDL cholesterol, nmol/L	1.6 (0.5)	1.4 (0.4)	1.2 (0.4)	< 0.001
LDL cholesterol, nmol/L	2.9 (1.4)	3.7 (1.5)	2.9 (1.0)	0.006
Triglycerides, nmol/L	0.8 (0.8)	1.2 (0.5)	1.5 (1.0)	< 0.001
Glucose, nmol/L	4.7 (0.70)	5.0 (0.7)	5.2 (0.8)	< 0.001
AST, U/L	3.9 (0.7)	3.9 (2.2)	4.3 (1.9)	0.388
ALT, U/L	3.6 (1.3)	4.3 (2.4)	5.3 (3.5)	< 0.001
GGT, U/L	2.8 (3.1)	3.3 (2.5)	5.1 (6.3)	0.002

Results are reported as median (interquartile range) or percentage. Statistical analysis were performed using Kruskal–Wallis or Chi-squared tests

**Fig. 2:**
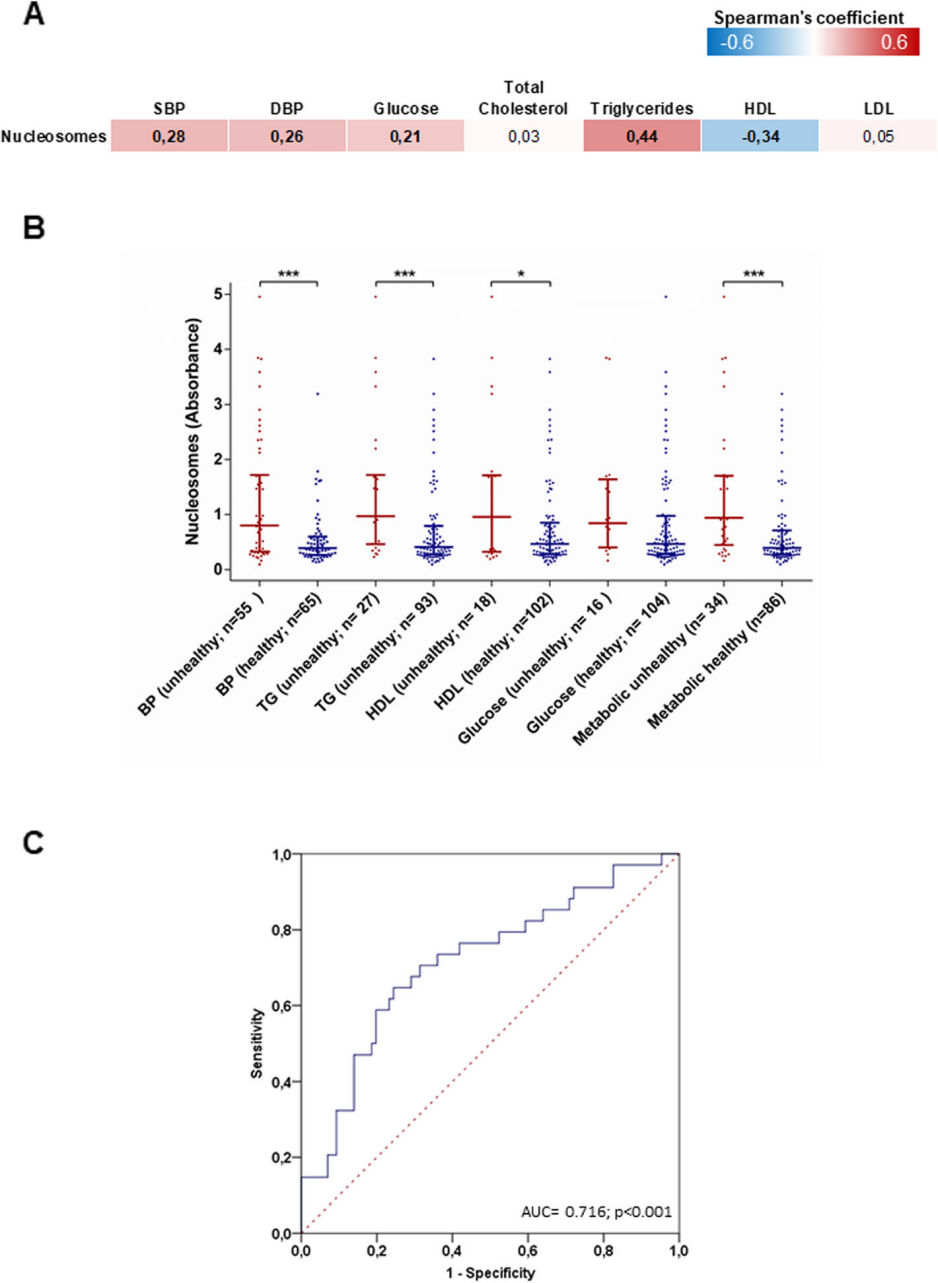
Associations between circulating nucleosome levels and parameters of metabolic health. Circulating nucleosomes are assayed by ELISA in Kardiovize participants (*n* = 120) and expressed as absorbance. **a** Correlation matrix between circulating nucleosome levels and cardiometabolic parameters. The data are reported as the Spearman’s correlation coefficient and those with a *p* value < 0.05 are indicated in bold. **b** Comparison of circulating nucleosome levels across metabolic health categories and its components. The data represent the median and interquartile range and are compared using the Mann-Whitney *U* test; ***p < 0.001; *p < 0.05. **c** ROC curve showing the ability of serum circulating nucleosomes to discriminate patients with poor metabolic health. *SBP* systolic blood pressure, *DBP* diastolic blood pressure, *TG* triglycerides

We next examined whether circulating nucleosome levels are also associated with metabolic health. Here, we found that serum nucleosome levels also positively correlated with systolic and diastolic blood pressure (*p* = 0.002 and *p* = 0.004, respectively), fasting glucose (*p* = 0.041), and triglyceride levels (*p* < 0.001), and negatively correlated with HDL cholesterol levels (*p* < 0.001) (Fig. 2a). Consistently, participants with poor metabolic health, in terms of abnormal blood pressure, triglyceride, and HDL cholesterol levels, showed higher serum circulating nucleosome levels than their metabolically healthy counterparts (*p* < 0.001; Fig. 2b). In particular, we observed that serum circulating nucleosome levels were higher in those with abnormal blood pressure (*p* < 0.001), triglycerides (*p* < 0.001), and HDL cholesterol (*p* = 0.039). Accordingly, ROC curve analysis demonstrated that serum nucleosome levels could discriminate patients with poor metabolic health with an AUC of 0.716 (95% CI = 0.612–0.821; *p* < 0.001; Fig. 2c). According to the definition of the minimum distance on the ROC curve from the (0, 1) point (distance: 0.184), the cut-off value of 0.714 serum nucleosome levels gave optimal detection of subjects with poor metabolic health (specificity = 75.6%; sensitivity = 64.7%). Taken together, these data imply that increased serum nucleosome levels are markers of poor metabolic health and high total and central adiposity.

**Fig. 3:**
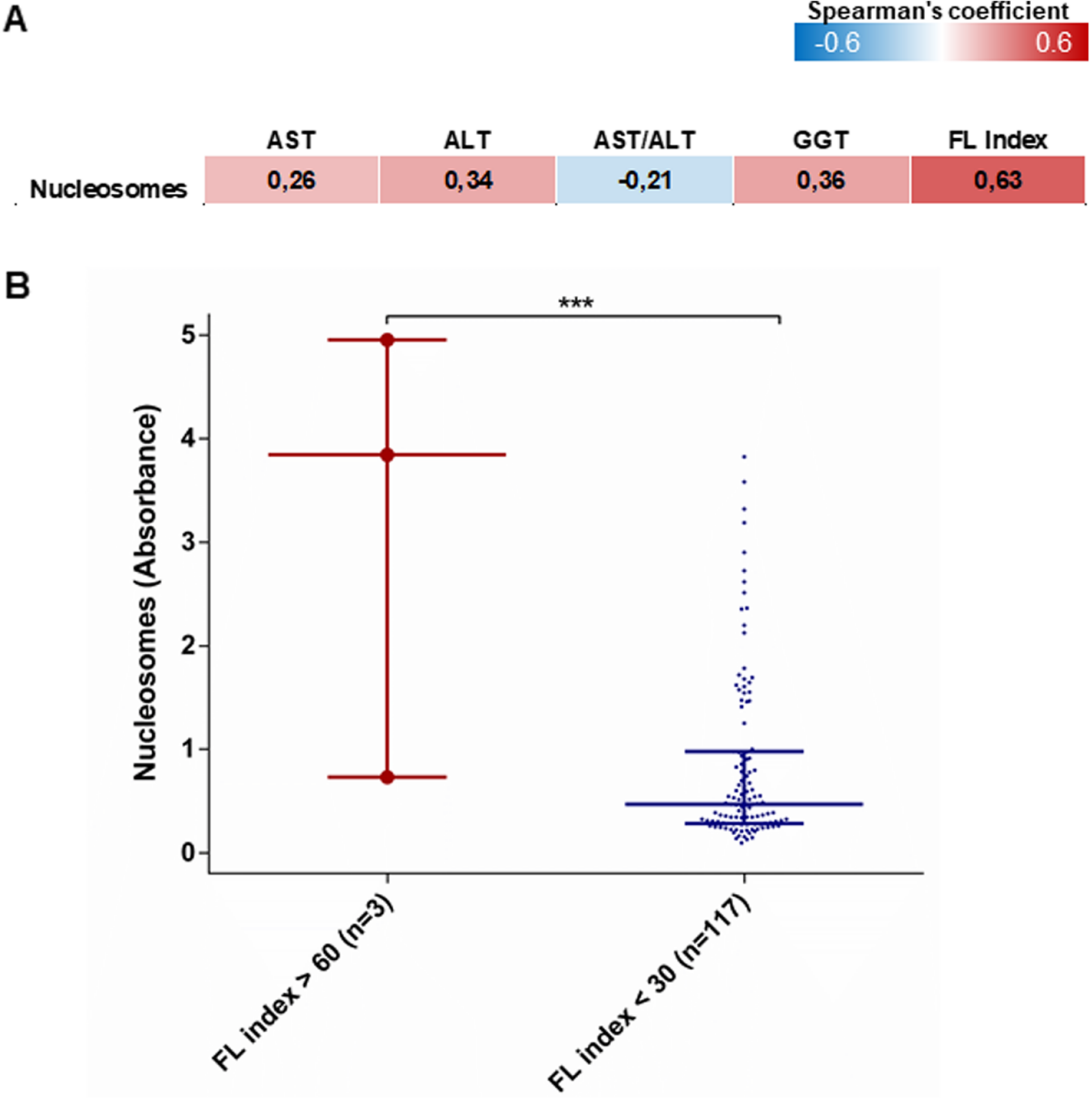
Association between circulating nucleosome levels, liver enzyme levels and fatty liver index. Circulating nucleosomes are assayed by ELISA in Kardiovize participants (*n* = 120) and expressed as absorbance. **a** Correlation matrix between circulating nucleosome levels and liver enzyme levels. The data are reported as the Spearman’s correlation coefficient and those with *p* value < 0.05 are indicated in bold. **b** Comparison of circulating nucleosome levels between fatty liver index categories. The data represent the median and interquartile range and are compared using the Mann-Whitney *U* test; ***p < 0.001. *AST* aspartate transaminase, *ALT* alanine aminotransferase, *GGT* gamma-glutamyltransferase, *FL* fatty liver

Obesity and metabolic abnormalities are associated with an increased risk of non-alcoholic fatty liver disease (NAFLD, or steatosis) and with mildly elevated circulating levels of liver enzymes [42, 43]. We thus evaluated the association between circulating nucleosome levels and liver enzyme levels in the 120 participants of the Kardiovize cohort. We found a positive, but weak correlation between nucleosome levels and AST (*p* = 0.004), ALT (*p* < 0.001), and GGT (*p* < 0.001) (Fig. 3a). Interestingly, however, we observed a strong positive correlation between serum circulating nucleosome levels and the FLI, meaning significantly higher nucleosome levels in obese patients at risk of hepatic steatosis (*p* < 0.001; Fig. 3b).

Altogether, these findings indicate that fatty liver associates with increased circulating nucleosome levels in the presence of obesity.

### Association of serum nucleosome levels with cardiovascular health

**Fig. 4:**
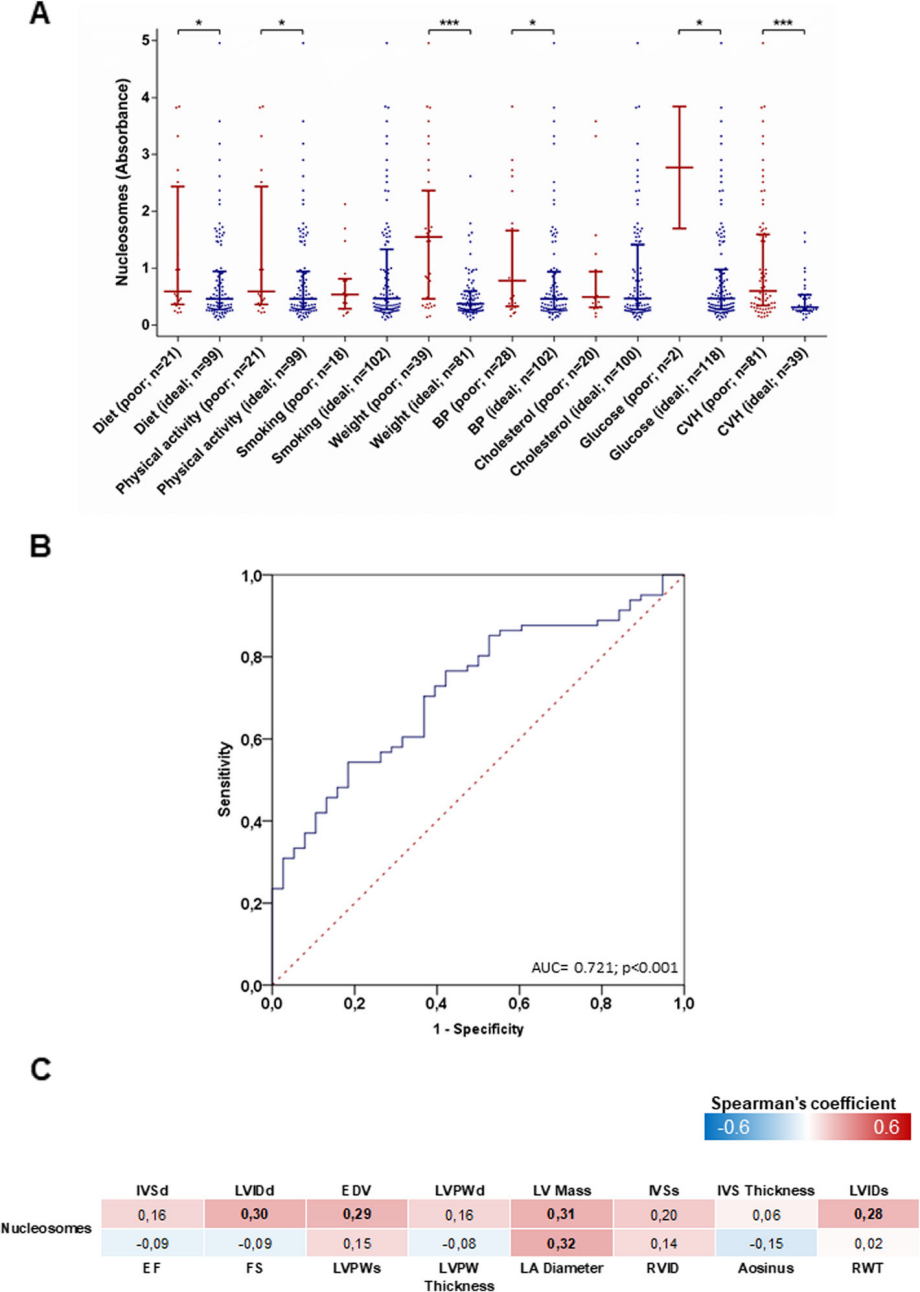
Association between circulating nucleosome levels and cardiovascular health (CVH). Circulating nucleosomes are assayed by ELISA in Kardiovize participants (*n* = 120) and expressed as absorbance. **a** Comparison of circulating nucleosome levels across CVH categories and its components. The data represent the median and interquartile range and are compared using the Mann-Whitney *U* test; ***p < 0.001; *p < 0.05. **b** ROC curve showing the ability of serum circulating nucleosomes to discriminate patients with poor CVH. **c** Correlation matrix between circulating nucleosome levels and echocardiographic parameters. The data are reported as the Spearman’s correlation coefficient and those with *p* value < 0.05 are indicated in bold. *BP* blood pressure, *CVH* cardiovascular health, *IVSd* interventricular septum thickness at end-diastole, *LVIDd* left ventricle end-diastolic diameter, *EDV* end-diastolic volume, *LV* left ventricular, *IVSs* interventricular septum thickness at end-systole, *LVIDd* left ventricle end-systolic diameter, *EF* ejection fraction, *FS* fractional shortening, *LVPWd* posterior wall thickness at end-diastole, *LA* left atrial, *RVID* right ventricle diameter, *Aosinus* aortic diameter at the sinus of Valsalva, *RWT* relative wall thickness

We next evaluated whether serum nucleosome levels are associated with CVH and its components, as defined by the AHA [24]. Interestingly, serum nucleosome levels significantly increased from ideal to poor categories of physical activity, diet, and blood glucose CVH components (Fig. 4a). Accordingly, subjects with poor CVH exhibited higher serum nucleosome levels than those with intermediate/ideal CVH (*p* < 0.001; Fig. 4a). In particular, we observed that serum nucleosome levels were higher in those with poor diet (*p* = 0.039), physical activity (*p* = 0.046), weight (*p* < 0.001), blood pressure (*p* = 0.043), and glucose (*p* = 0.049). The ROC curve analysis demonstrated that circulating nucleosomes levels could discriminate patients with poor CVH with an AUC of 0.721 (95% CI = 0.628–0.813; *p* < 0.001; Fig. 4b). According to the definition of the minimum distance on the ROC curve from the (0, 1) point (distance: 0.230), the cut-off value of 0.353 serum circulating nucleosome levels gave optimal detection of subjects with poor CVH (specificity = 60.5%; sensitivity = 72.8%). Interestingly, the detection improved when applying a stricter definition of poor CVH. In fact, we demonstrated that circulating nucleosomes levels could discriminate patients with CVH score ≤ 5 with an AUC of 0.811 (95% CI = 0.676–0.945; *p* = 0.002; Additional file 1: Figure S1). In line with this, the cut-off value of 1.473 for serum circulating nucleosome levels gave optimal detection of subjects with CVH score ≤ 5 (specificity = 82.7%; sensitivity = 71.8%).

Because CVH and its components are associated with cardiac remodeling [44], we finally evaluated the association between serum circulating nucleosome levels and echocardiographic parameters. Here, we found weak but significant correlation between increased serum nucleosomes and left ventricle end-diastolic diameter (LVIDd) (*p* = 0.013), end-diastolic volume (EDV) (*p* = 0.017), left ventricular (LV) mass (*p* = 0.011), left ventricle end-systolic diameter (LVIDs) (*p* = 0.021), and left atrium (LA) diameter (*p* = 0.009) (Fig. 4c). However, we found no significant differences in serum nucleosome levels between patients exhibiting cardiac remodeling and healthy controls (*p* = 0.466; Additional file 1: Figure S2). These data suggest that the serum nucleosomes associate with single hypertrophic indicators but do not predict pathological left ventricular geometry changes.

### HepG2 cells and adipocytes do not release nucleosomes into the extracellular environment in a free fatty acid-rich environment

Thus far, we have shown that circulating nucleosome levels are increased in obese individuals with poor CVH, consistent with findings from mouse models of obesity [19–21]. The tissue/cellular origin of these circulating nucleosomes in obesity, however, is unknown. We first aimed to ascertain whether circulating nucleosomes could be enriched specifically in trimethyl histone H3K4 (H3K4me3)—a marker of transcriptionally active chromatin that is increased in liver disorders and adipogenesis [45], and associated with a decreased life span [46]. We found no differences in circulating H3K4me3 levels by colorimetric assay across BMI categories (Additional file 1: Figure S3), implying that this post-translational histone H3 modification is not enriched in obese subjects.

**Fig. 5:**
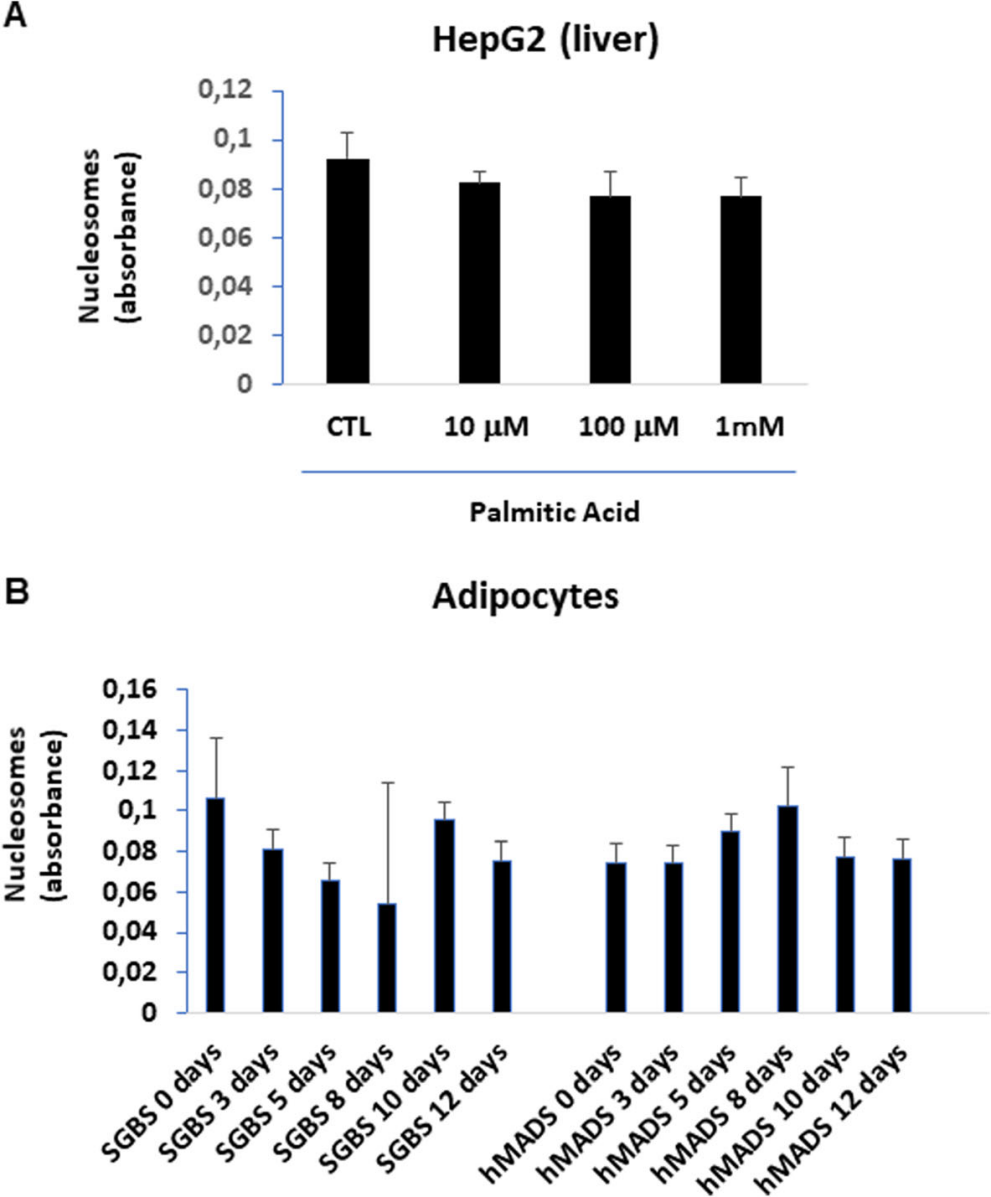
Palmitic acid does not trigger extracellular nucleosome production in cultured human hepatoma cells or adipocytes. **a** HepG2 cells were incubated for 24 h with palmitic acid at the indicated concentrations, before nucleosome absorbance was measured in the extracellular medium. **b** Human multipotent adipose-derived stem (hMADS) cells and pre-adipocytes from Simpson-Golabi-Behmel congenital syndrome (SGBS) were cultured and samples from cell culture supernatants were collected at 3, 5, 8, 10, and 12 days after adipocyte differentiation (see text), for subsequent measuring nucleosome concentration. The data represent the means ± SEM of three independent experiments

We subsequently sought to identify the cellular source of increased circulating nucleosomes. We hypothesized that either human hepatoma cells or adipocytes might produce and secrete nucleosomes when cultured in an obesogenic environment. These two cell types accumulate notable amounts of lipid droplets in the cytoplasm during obesity [47]. We first treated HepG2 cells with palmitic acid to induce hepatic lipid accumulation and lipotoxicity [36, 48] and then measured nucleosome secretion. Surprisingly, the treated cells did not secrete significant amounts of nucleosomes into the extracellular medium at any of the concentrations of palmitic acid used (0, 10 μM, 100 μM, 1 mM) (Fig. 5a).

As obesity is characterized by an excess in fat mass (either subcutaneous or visceral), we assessed the production of extracellular nucleosomes into the extracellular medium in two established models of human adipogenesis: hMADS cells [37, 38], and the pre-adipocytes from SGBS that are neither transformed nor immortalized, and are highly proliferative with a retained capacity for adipogenic differentiation [39, 49]. We collected cell culture supernatants at 3, 5, 8, 10, and 12 days after initiating adipocyte differentiation in these two cell lines. Again, we did not detect any significant differences in extracellular nucleosome levels at any of the differentiation stages tested compared to control (0 day, starting point), in either of the hMADS-derived or in SGBS-derived adipocytes (Fig. 5b). Taken together, these data suggest that the two major cell types storing fat during obesity do not release nucleosomes in the extracellular environment in obesogenic conditions.

### The effects of palmitic acid on neutrophil behavior and nucleosome release

**Fig. 6:**
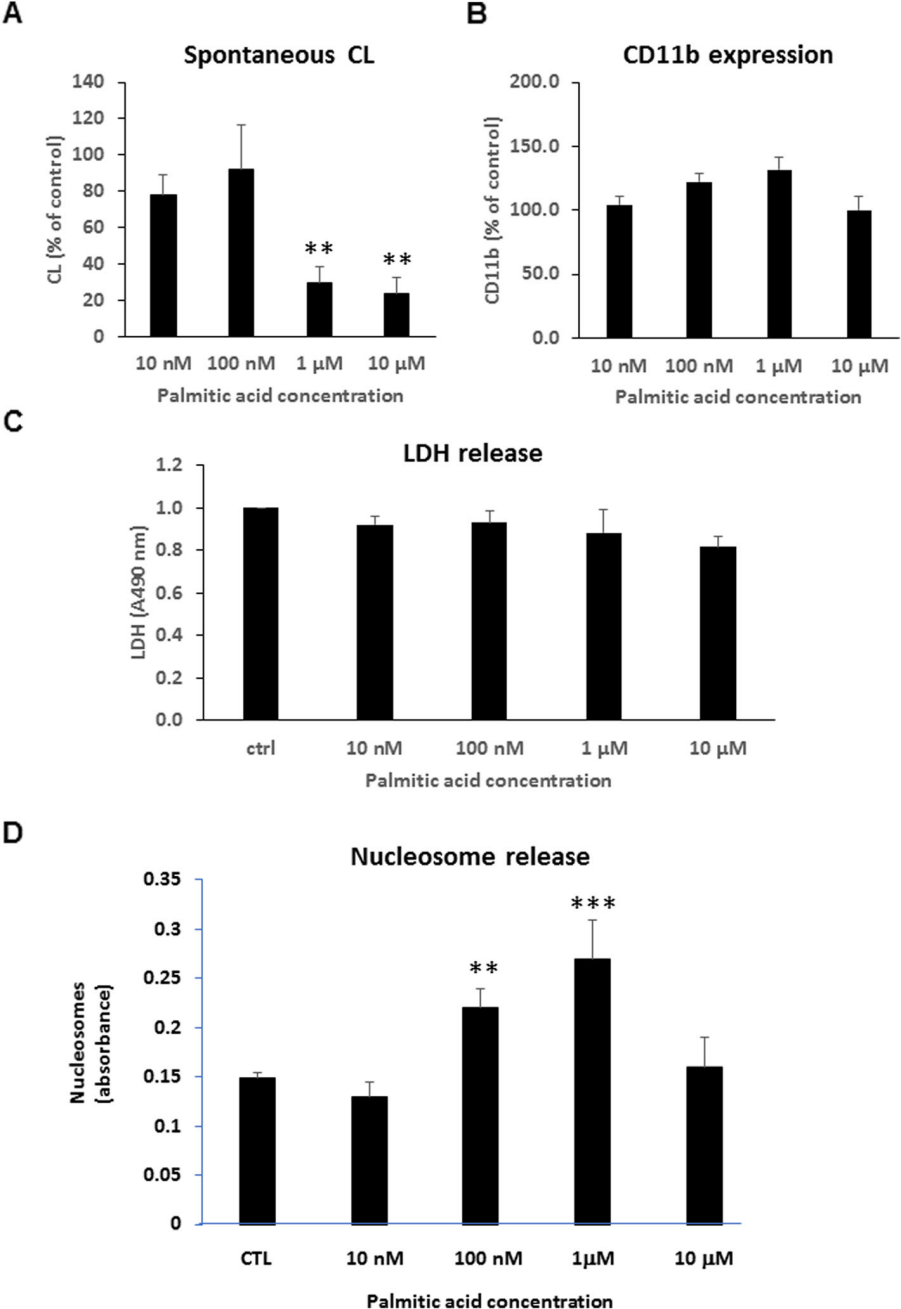
Exposure to free fatty acids triggers extracellular nucleosome production in human neutrophils. **a** Spontaneous production of reactive oxygen species by neutrophils expressed as the peak of the chemiluminescence response. *n* = 5. **b** Surface expression of CD11b on neutrophils. *n* = 3. **c** LDH concentration in neutrophil supernatants. *n* = 6. **d** Extracellular nucleosome production in human neutrophils. All data represent the means ± SEM and are compared using the Student’s *t* test; **p < 0.01; ***p < 0.001

In animals and patients with vein thrombosis, or conditions of systemic inflammation/neoplasia, elevated circulating nucleosomes associate with activated neutrophils [50, 51], and it has been suggested that the release of neutrophil-produced extracellular traps (NETs)—networks of extracellular fibers composed of DNA, histones, and other molecules that allow neutrophils to kill extracellular pathogens—might be a primary source of circulating nucleosomes, although the evidence has been elusive [52]. We sought thus to analyze the effect of an obesogenic environment on human neutrophils. We pre-incubated isolated neutrophils from healthy volunteers with palmitic acid for 4 h and then evaluated their resulting functions. We found that palmitic acid decreased neutrophil chemiluminescence, a measure of reactive oxygen species production, in a dose-dependent manner. This effect approached statistical significance for palmitic acid concentrations of 1 μM (*p* = 0.06) and 10 μM (*p* = 0.05) (Fig. 6a). Palmitic acid induced a trend in the increase in CD11b expression on the neutrophil surface, indicative of neutrophil activation, with concentrations up to 1 μM. No effect was observed with 10 μM palmitic acid, where CD11b expression was equal to that of untreated control (Fig. 6b).

In parallel, we performed a colorimetric assay based on lactate dehydrogenase (LDH) activity released from the cytosol of damaged cells into the supernatant, to quantify cell death and cell lysis. We found that exposing neutrophils to palmitic acid did not induce any cytotoxic effects, as none of the tested concentrations significantly increased LDH release into the culture supernatant (Fig. 6c). Thus, palmitic acid was proved not to be toxic to neutrophils in any of the concentrations. Interestingly, the neutrophils secreted significantly augmented amounts of nucleosomes into the extracellular medium upon exposure to 100 nm or to 1 μM of palmitic acid (Fig. 6d). The greatest effect was observed at the latter concentration, when neutrophils did not exhibit activation markers (Fig. 6a, b). Together, these data demonstrate that at non-cytotoxic concentrations of the most common saturated fatty acid in the serum of obese subjects, palmitic acid, nucleosome release is triggered from human neutrophils.

## Discussion

The present study aimed to analyze the role of intact nucleosomes—promising “liquid biopsies” released from dying cells into the blood circulation—as markers of MetS, CVH, and cardiac function. We show for the first time that circulating nucleosomes might function as universal markers of obesity, CVH, and MetS in a human cohort of 120 participants from the Kardiovize Brno 2030 study who did not present any other severe or acute illness. Interestingly, we also found a strong correlation to be between nucleosome levels and obesity and obesity-associated NAFLD (as detected by FLI).

Obesity, or a BMI > 30, is a growing issue imposing a devastating health and financial burden on affected individuals and society. Obesity is associated with high mortality rates driven by co-morbidities such as type 2 diabetes mellitus, MetS, and poor CVH, including CVD [53]. The CVH score, introduced in 2010 by the AHA, uses the levels of seven metrics (BMI, healthy diet, physical activity, smoking status, blood pressure, blood glucose, and total cholesterol) to identify from individuals with poor CVH, those at a high risk of CVD [30]. As CVD is now the leading cause of death globally [54], the identification of objective risk factors and easily measurable non-invasive (imaging, circulating markers) outcomes could have immediate relevance for public health strategies. Among developed countries, CVD has the highest incidence in Eastern and Central Europe [55]. To better understand the prevalence of cardiovascular risk factors in Central Europe, we established the Kardiovize Brno 2030 study, in which we recruited 2160 individuals to investigate the complex associations between CVD risk factors and outcomes [22].

One such novel risk factor that could be readily measured in affected patients are nucleosomes. Nucleosomes are elevated and serve as diagnostic/prognostic markers in cancer, stroke, trauma, and sepsis [3, 4, 6–9]. In addition, circulating nucleosomes are elevated in the plasma of mice fed a high-fat diet [19, 20] and in patients with acute pancreatitis with a high BMI [21]. We found that circulating nucleosomes presented moderate significant positive correlations with poor CVH score overall, and with single components of the CVH score, including high blood pressure, high triglyceride levels, weight, poor diet, poor physical activity, and high glucose levels. We found a negative correlation between circulating nucleosomes and HDL levels and no correlations with LDL levels, smoking status, or cholesterol levels. Of note, LDL and cholesterol levels are MetS markers independent of obesity [56], and the relationship between smoking and obesity is controversial and inconclusive [57].

Obesity is mainly characterized by an increase in adipose tissue, which contains different cell types, predominantly adipocytes, plus a minor stromal vascular fraction composed of fibroblasts, preadipocytes, vascular endothelial cells, and immune cells (macrophages, lymphocytes). Previous studies on obese animal models or patients have not investigated whether increased circulating nucleosome levels are derived from adipose tissue or specifically, adipocytes [19–21]. Here, we failed to detect a time-dependent dynamic change in the release of nucleosomes in the extracellular environment upon adipocyte differentiation, using two well-established models of human adipogenesis: human multipotent adipose-derived stem cells and the pre-adipocytes from SGBS [37, 38, 58, 59]. Interestingly, this finding suggests that nucleosome release in overweight/obese patients is not occurring during fat accumulation in adipocytes, and thus that the adipose tissue might not be the source of increased circulating nucleosomes in obesity. We also found that in vitro incubation of HepG2 cells with various concentration of saturated palmitic acid, triggering lipid accumulation [48], failed to liberate nucleosomes in the extracellular medium.

Limitations of these in vitro experiments that remain to be addressed, however, include the fact that (i) they do not provide information about the cross talk between different cell types, namely adipose tissue vs liver or inflammatory/immune cells; (ii) HepG2 cells are dissimilar to primary hepatocytes because they are immortalized and display large scale genome rearrangements and lower expression of some metabolic activities [60].

In vivo, lipid accumulation triggers IL-8 production by hepatocytes [58], which is a well-established inducer of neutrophil chemotaxis. Activated neutrophils propend to expel de-condensed chromatin embedded with inflammatory proteins, known as NETs, which are important in inflammation [61]. A recent study found elevated levels of NETs in mice and patients with non-alcoholic steatohepatitis (NASH) marker in serum of patients with NASH, and showed that elevated free fatty acids stimulate NET formation in vitro [62]. How neutrophil infiltration affects NAFLD progression in the presence of obesity, however, is still unclear.

In terms of the diagnostic value of liquid biopsies, anti-nucleosome antibodies have been shown to be superior to anti-DNA antibodies in detecting autoimmune diseases [10]. The diagnostic value of nucleosomes in human acute conditions might, however, be limited because diseases associated with accelerated cell death, such as ischemia, trauma, sepsis, and cancer, are also associated with elevated circulating cell free levels of nucleosomes [63]. Our studies suggest that even in the absence of acute illness, the clinical performance of the cell-free nucleosome is promising for detecting the deteriorating cardio-metabolic health associated with obesity, and offers a potential complementary, non-invasive, and universal diagnostic approach. Indeed, the predictive value of circulating nucleosomes for MetS and cardiovascular health was comparable, if not greater, to that reported for traditional adipose and metabolic markers [64].

Based on these results, we believe that further studies with larger numbers of patients are now warranted to confirm and validate the usefulness of circulating nucleosome epigenetic biomarkers in patients with poor CVH and MetS.

## Supplementary information

**Additional file 1: Figure S1.** ROC curve showing the ability of serum circulating nucleosomes to discriminate patients with CVH score ≤ 5 (AUC = 0.811; 95% CI = 0.676–0.945; *p* = 0.002). **Figure S2.** Circulating nucleosome levels are stable between patients with and without abnormal cardiac remodeling. Circulating nucleosomes are assayed by ELISA in Kardiovize participants (*n* = 120) and expressed as absorbance. The data represent the median and interquartile range and are compared using the Mann-Whitney U test. **Figure S3.** Circulating trimethyl histone H3K4 (M3K4m3) levels are stable across body mass index categories. The data represent the median and interquartile range and are compared using the Mann-Whitney *U* test. **Table S1.** Echocardiographic parameters of Kardiovize participants according to BMI categories.

## Data Availability

The datasets used and/or analyzed during the current study are available from the corresponding author on reasonable request.

## Abbreviations

AHA: American Heart Association
ATCC: American Type Culture Collection
AUC: Area Under the Curve
BMI: Body mass index
CI: Confidence Interval
CVD: Cardiovascular disease
CVH: Cardiovascular health
EDV: End-diastolic volume
FLI: Fatty liver index
GGT: Gamma-glutamyltransferase
hMADS: Human Multipotent Adipose-Derived Stem
LA: Left atrium
LDH: Lactate dehydrogenase
LV: Left ventricular
LVIDd: Left ventricle end-diastolic diameter
LVIDs: Left ventricle end-systolic diameter
MetS: Metabolic syndrome
NAFLD: Non-alcoholic fatty liver disease
NASH: Non-alcoholic steatohepatitis
NETs: Neutrophil-produced extracellular traps
ROC: Receiver-Operating Characteristic
SGBS: Simpson-Golabi-Behmel syndrome
T2DM: Diabetes mellitus
