# Supplementary material for: Obesity-induced nucleosome release predicts poor cardio-metabolic health
Source: Clin Epigenetics. 2019 Dec 31;12:2. doi: 10.1186/s13148-019-0797-8 (PMC6938639; doi:10.1186/s13148-019-0797-8)
Supplement: Supplementary file 1 — Additional file 1: Figure S1. ROC curve showing the ability of serum circulating nucleosomes to discriminate patients with CVH score ≤ 5 (AUC = 0.811; 95% CI = 0.676–0.945; p = 0.002). Figure S2. Circulating nucleosome levels are stable between patients with and without abnormal cardiac remodeling. Circulating nucleosomes are assayed by ELISA in Kardiovize participants (n = 120) and expressed as absorbance. The data represent the median and interquartile range and are compared using the Mann-Whitney U test. Figure S3. Circulating trimethyl histone H3K4 (M3K4m3) levels are stable across body mass index categories. The data represent the median and interquartile range and are compared using the Mann-Whitney U test. Table S1. Echocardiographic parameters of Kardiovize participants according to BMI categories. [file 13148_2019_797_MOESM1_ESM.docx]

**Table S1.** Echocardiographic parameters of Kardiovize participants according to BMI categories

| **Echocardiographic parameters** | **Normal weight**  **(n=46)** | **Overweight**  **(n=34)** | **Obese**  **(n=40)** | **p-value** |
| --- | --- | --- | --- | --- |
| **IVSd** | 1.0 (0.2) | 0.9 (0.2) | 1.2 (0.2) | 0.060 |
| **LVIDd** | 5.0 (0.9) | 4.3 (0.3) | 4.9 (0.4) | 0.470 |
| **EDV** | 119.5 (48.3) | 81.0 (12.0) | 115.0 (18.1) | 0.480 |
| **LVPWd** | 0.9 (0.1) | 0.9 (0.2) | 0.9 (0.2) | 0.700 |
| **LVM** | 139.9 (62.3) | 147.9 (33.6) | 198.2 (55.5) | 0.070 |
| **IVSs** | 1.4 (0.3) | 1.2 (0.2) | 1.5 (0.3) | 0.030 |
| **IVS thickness** | 40.0 (35.5) | 35.0 (20.0) | 30.0 (23.0) | 0.731 |
| **LVIDs** | 3.3 (0.7) | 2.7 (0.4) | 3.1(0.3) | 0.309 |
| **EF** | 72.5 (6.5) | 75.0 (9.5) | 76.0 (7.0) | 0.534 |
| **FS** | 35.0 (5.5) | 37.0 (9.0) | 38.0 (5.0) | 0.516 |
| **LVPWs** | 1.5 (0.2) | 1.3 (0.2) | 1.5 (0.2) | 0.050 |
| **LVPW thickness** | 69.5 (47.5) | 33.0 (26.5) | 49.0 (33.0) | 0.327 |
| **LA diameter** | 3.4 (0.7) | 3.6 (0.6) | 3.5 (0.7) | 0.190 |
| **RVID** | 3.5 (0.7) | 3.5 (0.7) | 3.9 (0.6) | 0.206 |
| **Aosinus** | 3.2 (0.5) | 2.7 (0.6) | 3.0 (0.5) | 0.053 |
| **RWT** | 0.3 (0.1) | 0.4 (0.1) | 0.4 (0.1) | 0.669 |

Results are reported as median (Interquartile range). Statistical analysis were performed using Kruskal–Wallis test.

**Figure S1.** ROC curve showing the ability of serum circulating nucleosomes to discriminate patients with CVH score ≤ 5 (AUC= 0.811; 95% CI = 0.676–0.945; p=0.002).


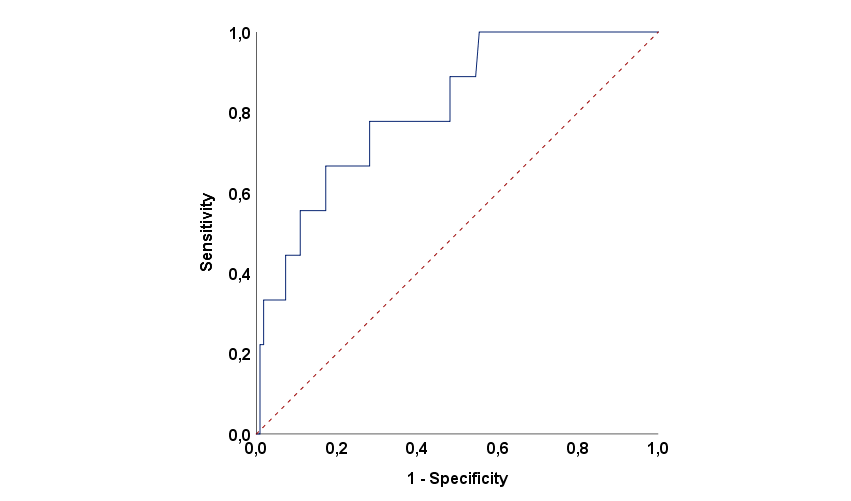


**Figure S2.** Circulating nucleosome levels are stable between patients with and without abnormal cardiac remodeling. Circulating nucleosomes are assayed by ELISA in Kardiovize participants (n=120) and expressed as absorbance. The data represent the median and interquartile range and are compared using the Mann-Whitney U test.





**Figure S3.** Circulating trimethyl histone H3K4 (M3K4m3) levels are stable across body mass index categories. The data represent the median and interquartile range and are compared using the Mann-Whitney U test.
